# Supplementary material for: Cycles of gene expression and genome response during mammalian tissue regeneration
Source: Epigenetics Chromatin. 2018 Sep 12;11:52. doi: 10.1186/s13072-018-0222-0 (PMC6134763; doi:10.1186/s13072-018-0222-0)
Supplement: Supplementary file 1 — Additional file 1: Fig. S1. (A) Description of the mouse liver regeneration stages, collection time points of liver samples and the food and light conditions of the mice. Adapted from [10]. X = eXcised liver hepatectomy; C = controls; S = sham surgeries; W = week. (B) Number of replicates per experimental condition and the associated RNA-Seq and ChIP-Seq experiments performed. In a preliminary PH analysis (Series 1), we performed ChIP-Seq on pools of three livers at the post-PH time points indicated and no sham surgeries. Subsequently, we performed Series 2 with the full set of time points and selected sham surgeries, with RNA-Seq analyses of generally three individual livers and, where indicated, ChIP-Seq on pools of three livers. Note that Series 2 was performed in three separate time periods (Series 2.1, 2.2, 2.3); for two samples, X20 and X36, duplicate samples were prepared in Series 2.1 and 2.2. The samples from Series 2.1 were used in the data shown and discussed in the text. The Series 1 60-h sample was used for the K36me2 versus H3K36me3 study in Fig. 6. (C) Correlation of three triplicates from each of Series 2.1, 2.2, and 2.3 (nine samples total) at time C0. The replicates are indicated on the diagonal. Above the diagonal, pairwise scatterplots display the similarity between replicates, and below the diagonal, each pairwise Pearson coefficient is indicated. Correlations within experimental series (Pearson correlation coefficients of at least 0.98) show slightly better correlation coefficients than among series (Pearson correlation coefficient of at least 0.94). (D) Two-dimensional plot displaying the coordinates of the collected samples in the PC1 and PC2 of the PCA using the set of 12,025 expressed genes as shown in Fig. 1b, but with the standard deviations in PC1 and PC2 of the replicates for each condition displayed as ovals. [file 13072_2018_222_MOESM1_ESM.pdf]

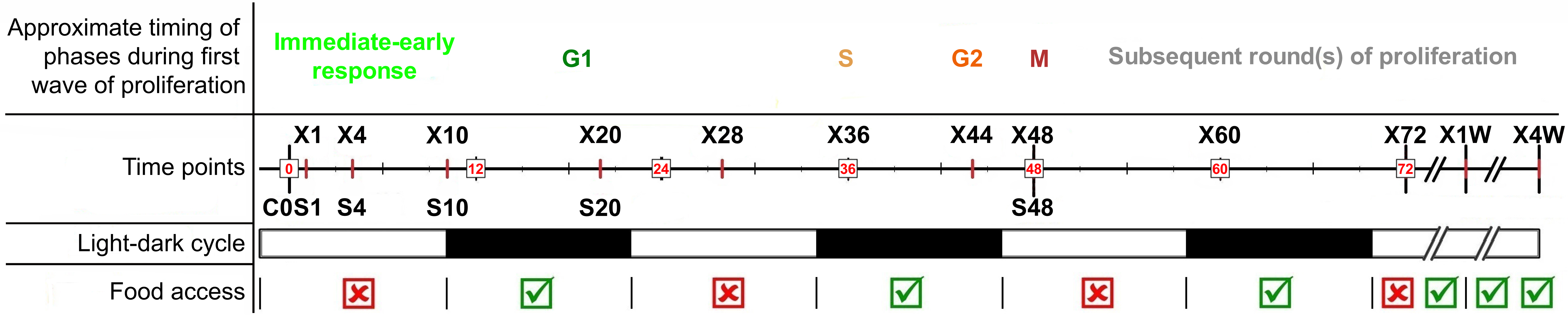
Approximate timing of phases during first wave of proliferation
Immediate-early response
G1
S
G2
M
Subsequent round(s) of proliferation
Time points
X1
X4
X10
X20
X28
X36
X44
X48
X60
X72
X1W
X4W
0
12
24
36
48
60
72
C0
S1
S4
S10
S20
S48
Light-dark cycle
Food access

Sup. Fig 1A

Sample	Series 1		Series 2.1		Series 2.2		Series 2.3	
	ChIP-seq	RNA-seq	ChIP-seq	RNA-seq	ChIP-seq	RNA-seq	ChIP-seq	RNA-seq
**C0**	1			3	1	3		3
**C4**								3
**C4W**						2		
**S1**				3	1	3		
**S4**				3	1	2		
**S10**					1	3		
**S20**					1	3		
**S48**				3	1			
**X1**	1			3	1	3		
**X4**				3	1	3		
**X10**	1			3	1	3		
**X20**	1		1	3	1	3		
**X28**	1		1	3				
**X36**	1		1	3	1	3		
**X44**	1		1	3				
**X48**	1		1	5				
**X60**	1		1	3				
**X72**			1	3				
**X1W**			1	3				
**X4W**						3		

Sup. Fig 1B

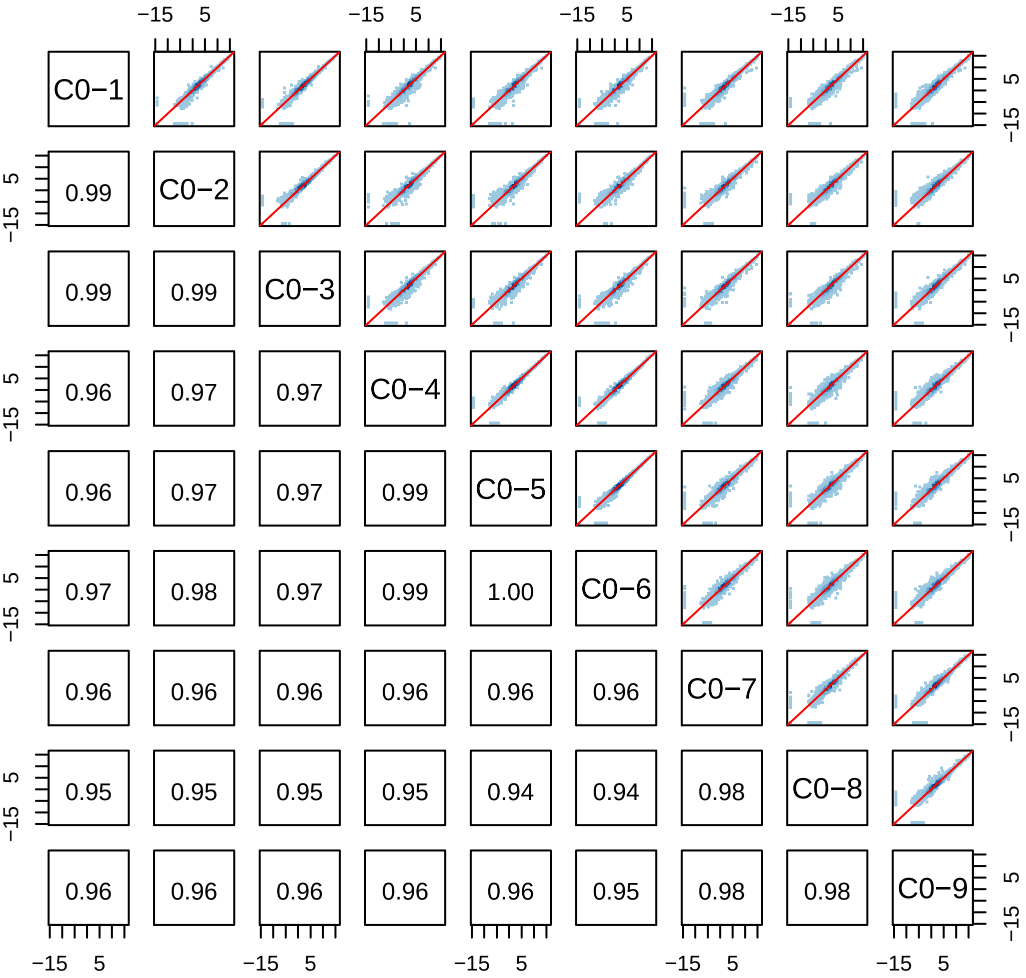
C0−1
C0−2
C0−3
C0−4
C0−5
C0−6
C0−7
C0−8
C0−9
0.99
0.99
0.99
0.96
0.97
0.97
0.96
0.97
0.97
0.99
0.97
0.98
0.97
0.99
1.00
0.96
0.96
0.96
0.96
0.96
0.96
0.95
0.95
0.95
0.95
0.94
0.94
0.98
0.96
0.96
0.96
0.96
0.96
0.95
0.98
0.98
−15
5

Sup. Fig. 1C

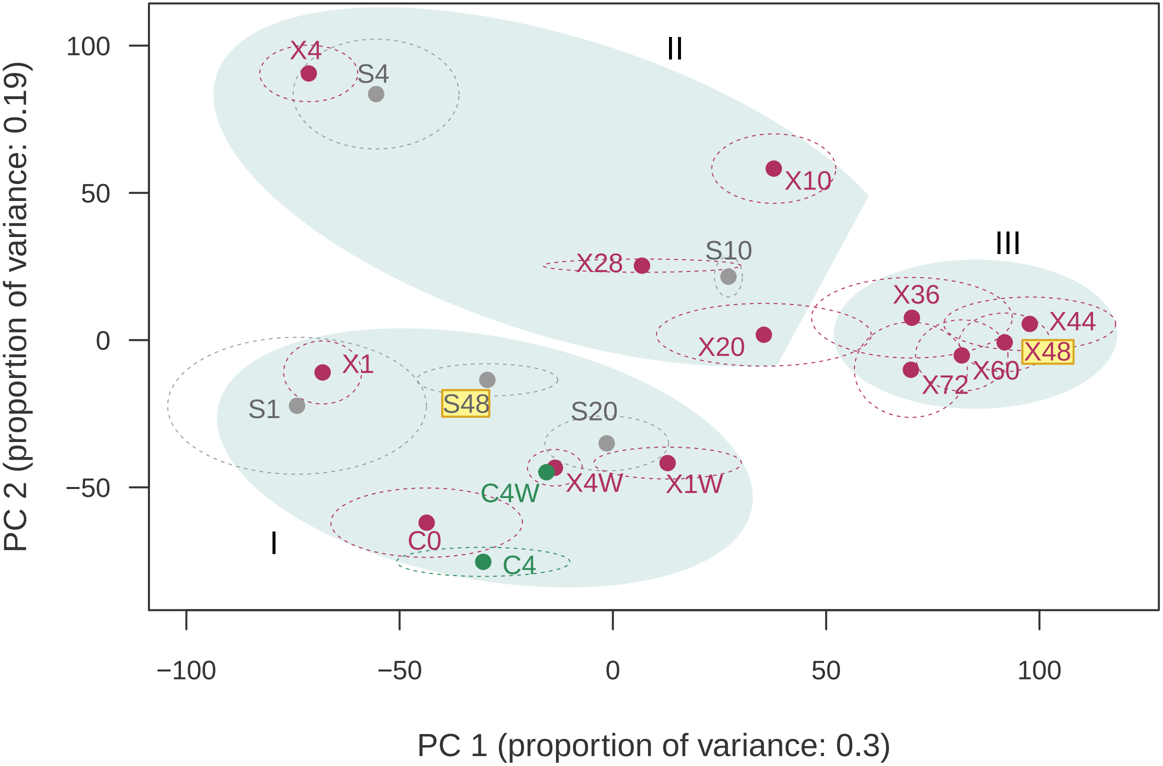

PC 2 (proportion of variance: 0.19)
PC 1 (proportion of variance: 0.3)
100
50
0
−50
−100
−50
0
50
100
I
II
III
X4
S4
X10
X28
S10
X36
X44
X20
X48
X60
X72
X1
S1
S48
S20
X4W
X1W
C4W
C0
C4

Sup. Fig. 1D
