# Supplementary material for: Cycles of gene expression and genome response during mammalian tissue regeneration
Source: Epigenetics Chromatin. 2018 Sep 12;11:52. doi: 10.1186/s13072-018-0222-0 (PMC6134763; doi:10.1186/s13072-018-0222-0)
Supplement: Supplementary file 2 — Additional file 2. Supplemental Methods and References. [file 13072_2018_222_MOESM2_ESM.pdf]

Supplemental Methods

Animals and partial hepatectomy (PH) and sham operations

Male C57/BL6 mice were 12–14 weeks old at the time of PH or sham surgery. They had been entrained for two weeks with a ZT0–ZT12 light and ZT12–ZT24 dark circadian cycle followed by two further weeks on cycles of ZT0–ZT12 light with fasting and ZT12–ZT24 dark with feeding. Groups of three mice were subjected to 2/3 PH as described [1] under isoflurane anesthesia at ZT2, and sacrificed together by cervical dislocation at 1, 4, 10, 20, 28, 36, 44, 48, 60, 72 h and one and four weeks post-surgery. Sham-operated controls were subjected to laparotomy under the same conditions and livers were collected at 1, 4, 10, 20 and 48 h after the operation.

RNA-Seq library preparation and sequencing

Poly(A)-containing RNA from individual livers was selected using the miRNeasy Mini Kit (Qiagen 217004 (50)) and used for RNA-seq without spike in. Strand-specific libraries were prepared with the TruSeq Stranded mRNA Library Prep kit from Illumina. The fragment ends (100 nucleotides) were sequenced with single-end sequencing technology from HiSeq 2100 (Illumina).

Chromatin immunoprecipitation (ChIP)

The ChIP protocol was adapted from [2]. Three livers per time point were collected, immediately individually homogenized in 4 ml 1 x PBS with 1% formaldehyde, and incubated for10 min at room temperature for cross linking. Cross-linking reactions were stopped by the addition of 25 ml of ice-cold 2.2 M sucrose buffer (150 mM glycine, 10 mM HEPES pH 7.6, 15 mM KCl, 2 mM EDTA, 0.15 mM spermine, 0.5 mM spermidine, 0.5 mM DTT and 0.5 mM PMSF). The resulting homogenate was layered onto a 10 ml cushion of ice-cold 2.05 M sucrose buffer as

above but including 10% glycerol and 125 mM glycine and centrifuged for 1 h at 24,000 rpm (100,000g) at 4 °C in a Beckmann SW28 rotor. The nuclear pellet was re-suspended sequentially twice in 0.7 ml of ice-cold Buffer A (20 mM Tris, pH 7.5, 150 mM NaCl, 2 mM EDTA), transferred to a 1.5-ml microcentrifuge tube and pelleted at 2000 rpm in a microcentrifuge for 30 sec. The pellet was re-suspended in 0.5 ml (20% glycerol, 20 mM HEPES pH 7.6, 0.2 mM EDTA, 2 mM DTT, 100 mM KCl), snap frozen and stored overnight at -70°C.

After thawing, the nuclei were re-pelleted as above and, for each time point collection, the pellets from each of triplicate livers were re-suspended, pooled with a total of 0.7 ml ice-cold Buffer A and re-pelleted in a microfuge for 1 min at 2000 rpm as above. The nuclei were subsequently re-suspended in 1.2 ml of Nuclear Lysis Buffer (NLB; 50 mM Tris, pH 8.1, 10 mM EDTA, 1% SDS, 0.15 mM spermine, 0.5 mM spermidine, 0.5 mM DTT and 0.5 mM PMSF) and left on ice for 10 min. After transfer to a 5 ml snap cap tube and addition of 0.7 ml Immunoprecipitation Dilution Buffer (IPDB; 20mM Tris-HCl pH 8.1, 150 mM NaCl, 2 mM EDTA, 1% Triton X-100, 0.01% SDS, 50 μg/ml PMSF, 1 μg/ml leupeptin, 0.15 mM, spermine, 0.5 mM spermidine), the samples were sonicated with a Bioruptor-Pico (Diagenode) to a size range of 300–1000 bp. Sheared chromatin was transferred to a 2 ml microfuge tube, centrifuged at maximum velocity for 5 min at 4°C, and transferred to 4.1 ml IPDB (6 ml total 1:4 NLB:IPDB solution). Prior to immunoprecipitation, the chromatin was pre-cleared with 100 µl of naive serum for 1 h at 4°C on a rotating wheel, followed by addition of 200 µl of a 50% protein A-agarose PBS slurry (Roche). After incubation for 3 h at 4°C on a rotating wheel, the samples were centrifuged in a microfuge for 2 min at 3000 rpm. The supernatant was collected and DNA content quantified.

For each immunoprecipitation, 19 μg sonicated mouse liver DNA was mixed with 1 μg sonicated human HeLa cell DNA. (Note that although not used in the final computational analysis, the samples were spiked with 5% of human HeLa-cell chromatin as an internal control for ChIP quantitation [3]). For immunoprecipitation, chromatin was diluted to 1.2 ml IP buffer

(26mM Tris-HCl pH 8.1, 120 mM NaCl, 2.6 mM EDTA, 0,8% Triton X-100, 0.206% SDS, 50 μg/ml PMSF, 1 μg/ml leupeptin, 0.15 mM, spermine, 0.5 mM spermidine) and incubated overnight at 4°C on a rotating wheel with the following antibodies: anti-RPB2 (Santa Cruz Biotechnology, sc-673-18), anti-H3K4me3 (Abcam, ab8580), anti-H3K36me3 (Abcam, ab9050) and anti-H3K36me2 (Upstate 07-274), 40 µl of 50% protein-A bead slurry was added to the antibody/chromatin mix and incubated for a further 3 h at 4°C on a rotating wheel. The beads were then washed twice with 1 ml of Immunoprecipitation Wash Buffer 1 (IPWB1; 20 mM Tris-HCl pH 8.1, 50 mM NaCl, 2 mM EDTA, 1% Triton X-100, 0.1% SDS) and centrifuged for 2 min at 3000 rpm at 4°C. The bead pellet was subsequently washed with 1 ml of IPWB2 (10 mM Tris-HCL pH 8.1, 250 mM LiCl, 1 mM EDTA, 1% NP-40, 1% sodium deoxycholate) and centrifuged for 2 min at 3000 rpm at 4°C. The beads were similarly further washed twice with 1 ml of 1x TE pH 8.0 and centrifuged for 2 min at 3000 rpm at 4°C. Protein–DNA complexes were eluted from the beads and the purified DNA along with corresponding input (Input) cross-linked samples were de-cross-linked, and treated with RNase A followed by proteinase K, as described [4]. The DNA concentration was determined by fluorometry with the Qubit system (Invitrogen). A total of 10 ng DNA was used for the ultra-high-throughput DNA sequencing library preparation.

ChIP-Seq library preparation and ultra-high-throughput sequencing

Paired-end sequencing libraries were prepared with the 'MicroPlex Library Preparation' kit, following the instructions of the manufacturer (Diagenode, catalog no C05010011). No electrophoretic size selection was performed and the chromatin was amplified with 14 cycles of polymerase chain reaction (PCR). 100 nucleotides at both fragment ends were sequenced with the paired-end sequencing technology from HiSeq 2100 (Illumina).

RNA-Seq data preparation and quantification

Sequenced reads of low quality were discarded (i.e., quality of the first 25 bp lower than Q20, as specified by Illumina). Adapters were removed with the Cutadapt tool [5]. Sequences shorter than 40 bp and sequences with low complexity were discarded with the tool ‘prinseq- lite’ [6]. For the removal of low complexity sequences the method ‘dust’ was used. The selected reads were mapped with Tophat 2.0.13 [7] in combination with Bowtie2 [8] Reads were first mapped onto coding sequences annotated in the mm9 mouse annotation and unmapped reads were aligned onto the genome. The resulting mapped reads were sorted with samtools 0.1.19 [9] and were assigned to genes by using the ‘featureCounts’ function from the ‘Rsubread’ R package [10]. Reads corresponding to the synthesized RNA strand and mapping within genes were assigned to the corresponding gene. Reads mapping uniquely were given a value of 1 read, whereas reads mapping to multiple genomic positions were assigned a score of 1 read divided by the number of different mapped positions. Finally, Reads Per Kilobase of transcript per Million mapped reads (RPKM) were calculated and used for computational analyses. All the handling of the data was done with the UNIX shell, Perl and the R software [11].

ChIP-Seq data preparation and quantification

The terminal 50 bp of the two sequenced paired-ends were mapped onto the mouse (mm9) and human (hg19) genomes with Elandv2e. Only fragments with good sequencing quality (i.e., quality of the first 25 bp higher than Q20, as specified by Illumina) for both ends and mapping on unique genomic locations were retained. From the genomic location of the ends of paired sequences, we could deduce the fragment size. For further analysis, fragment sizes between 50 and 500 bp were taken and only one copy of fragments with identical ends was kept. The density of retrieved fragments was displayed by showing the central deduced 50 bp of the sequenced fragments.

Using the Ensembl 67/NCBI 37 transcription-unit annotation, Pol II density at promoter regions was defined using the central 50 bp of Pol II ChIP fragments from 50– 500bp long

within +/- 250 bp of annotated TSS. Quantitation of H3K4me3 densities was +/- 500 bp around the same TSS selected for Pol II measurement. Pol II and H3K36me3 quantitation in the body of annotated transcription units was done from 500 bp 3' of the TSS to 2 kb 3' of the poly(A) signal in the sense of the synthesized RNA. The same series of quantitation was done on the Input samples. All quantifications were scaled to the number of fragments analyzed per library. 500 pseudo-counts were added prior to all density calculations to stabilize the variance of low scores. Log2 ratios between ChIP and Input quantifications were calculated. To correct for the difference of signal between genic and intergenic regions, the log2 ratios between ChIP and Input quantifications in genic regions were normalized to the mean log2 ratio obtained from intergenic regions positioned 2 kb or more away from any annotated transcription unit.

Among the multiple transcription units associated with a given gene, only the one containing the maximum promoter-associated Pol II occupancy was used for the analysis of Pol II body, H3K4me3 and H3K36me3. All the handling of the data was done with the UNIX shell, Perl and the R software [11].

Identification of genes expressed and differentially expressed post PH

The identification of significant gene expression was done on each library by implementing the method proposed by [12]. The maximum threshold obtained across libraries was taken as final threshold (log2(RPKM)=-1.5). The final number of genes expressed in at least one library was 12,025. The expressed genes were further classified into stable and changing expression compared to the resting liver using the edgeR library implemented on R [13]. Tag-wise dispersion was estimated and gene-wise exact tests were computed to identify differences in means of the replicates between time points, defined as negative binomial random variables. P-values were corrected as described [14]. Only corrected p-values lower than 1×10^{-7} were used and log2 fold-changes higher than 0.5 or lower than -0.5 retained.

Transcriptome clustering, PCA and differential gene expression clustering

Genes (12,025) expressed in at least one sample between 0 and 60 h were grouped based on their dynamics post PH between 0 to 4 weeks. For this analysis, the log2(RPKM) quantifications per replicate samples were averaged. The grouping of genes was done with a hierarchical clustering strategy implemented in R [11]. Hierarchical clustering was performed on the z-scores by using the Ward's minimized variance criterion in order to minimize the total variance within clusters [15]. The Ward's minimized variance criterion was used on Euclidean distances between pairs of samples. And the R library 'dendextend 0.17.1' was used to handle the resulting dendogram [16].

To summarize the variation in gene expression across samples, a PCA was performed. First, for each sample, z-scores were calculated from the the log2 RPKMs quantified on genes. A standard PCA was performed on the scaled quantifications.

For differential gene expression clustering, we used the PAM algorithm from the R 'cluster 1.14.4' library [17] on the changing Set 3 genes using 1 minus the Pearson correlation coefficient as the dissimilarity measure. The log2(RPKM) quantifications were centered and scaled to obtain z-scores. The clustering results were displayed using the 'ggplot 2.14.1' R library heatmap.2 function [18]. For the final display, clusters were sorted based on whether expression decreased (Bracket I in Figure 2A) or increased (Bracket II) post PH and then sequentially from top to bottom by the time when the preponderance of genes contained in the cluster began to exhibit an altered expression post PH.

Display of gene-expression profiles

Line plots were created with the profiles of RNA and Pol II log2 quantifications. For the Pol II quantifications both the proximal promoter and body of gene profiles are shown. Shaded lines or error bars are displayed through-out to describe the standard deviation across samples. All the plots were created using the 'ggplot2' library [19] used in the R statistical software [11].

Comparison of differentially expressed genes between post-PH and sham samples

The log2(RPKM) quantifications across replicate samples were averaged for each gene classified as differentially expressed post PH. A comparison was done at each time point where both sham and post-PH samples of RNA were collected. For the comparison, log2 ratios were calculated between the post-PH and sham averaged quantifications. Fold changes on genes with log2(RPKM)'s lower than 0 at both conditions were not included. Plots were created with the R statistical software [11].

Functional enrichment of gene lists and pathway annotation

The Webgestalt tool was used [20] to perform functional enrichment analyses and pathway annotation of gene lists. For these analyses, the GeneOntology database was consulted [21]. The p-values associated to GO Terms were corrected by the Benjamini method and only those terms with corrected p-value lower than 0.05 were kept. To reduce the redundancy of terms intrinsic to the GO database [22], the retrieved lists where summarized by using the REVIGO tool [23]. The list of GO terms with corrected p-value lower than 10-10 were used. The REVIGO algorithm was executed using SimRel as a measure of semantic similarity among GO terms and allowing a "Medium" similarity level to aggregate similar terms.

The annotation of cell-cycle genes was obtained from the KEGG database (entry mmu04110) [24]. Entrez gene identifiers associated to the KEGG cell cycle pathway annotation were converted to Ensembl identifiers by using the biomaRt tool in R [25, 26] that in turn accessed the org.Mm.eg.db 2.10.1 database [27]. To assign desired colors to the nodes in the cell cycle KEGG pathway an adaptation of the ‘pathview’ R package was used [28].

Density profiles of H3K36me2 and H3K36me3 accumulation on multiple genes at a one-nucleotide resolution

Nucleotide positions around the 3' end of the first internal exon of selected transcripts were

taken. The analyzed regions of Series 1 time point 60 h started at 4 kb upstream of the 3' end of the first internal exon and ended at 1 kb downstream. All the genomic regions were aligned from 5' to 3'. All the handling of genomic regions was done on the UNIX shell.

On the nucleotide positions, densities of the central 50 bp of sequenced fragments were quantified at the previously defined nucleotide positions. A matrix of quantifications per Transcription Unit (TU) was built and analyzed on the R statistical software [11]. Z-scores were calculated from the quantifications and displayed with the heatmap.2 function included on the ‘ggplots 2.14.1’ [18].

Supplemental References

1. Mitchell C, Willenbring H. A reproducible and well-tolerated method for 2/3 partial hepatectomy in mice. Nat Protoc. 2008;3:1167–70.

2. Le Martelot G, Canella D, Symul L, Migliavacca E, Gilardi F, Liechti R, et al. Genome-Wide RNA Polymerase II Profiles and RNA Accumulation Reveal Kinetics of Transcription and Associated Epigenetic Changes During Diurnal Cycles. PLoS Biol. 2012;10.

3. Bonhoure N, Bounova G, Bernasconi D, Praz V, Lammers F, Canella D, et al. Quantifying ChIP-seq data: A spiking method providing an internal reference for sample-to-sample normalization. Genome Res. 2014;24:1157–68.

4. O'Geen H, Nicolet CM, Blahnik K, Green R, Farnham PJ. Comparison of sample preparation methods for ChIP-chip assays. Biotechniques. 2006;41:577–80.

5. Martin M. Cutadapt removes adapter sequences from high-throughput sequencing reads. EMBnet.journal. 2011;17:10

6. Schmieder R, Edwards R. Quality control and preprocessing of metagenomic datasets. Bioinformatics. 2011;27:863–4.

7. Kim D, Pertea G, Trapnell C, Pimentel H, Kelley R, Salzberg SL. TopHat2: Accurate alignment of transcriptomes in the presence of insertions, deletions and gene fusions. Genome Biol. 2013;14.

8. Langmead B, Salzberg SL. Fast gapped-read alignment with Bowtie 2. Nat Methods. 2012;9:357–9.

9. Li H, Handsaker B, Wysoker A, Fennell T, Ruan J, Homer N, et al. The Sequence Alignment/Map format and SAMtools. Bioinformatics. 2009;25:2078–9.

10. Liao Y, Smyth GK, Shi W. The Subread aligner: Fast, accurate and scalable read mapping by seed-and-vote. Nucleic Acids Res. 2013;41.

11. Team R. R Development Core Team. R A Lang Environ Stat Comput. 2013;55:275–86.

12. Hart T, Komori HK, LaMere S, Podshivalova K, Salomon DR. Finding the active genes in deep RNA-seq gene expression studies. BMC Genomics. 2013;14.

13. Robinson MD, McCarthy DJ, Smyth GK. edgeR: a Bioconductor package for differential expression analysis of digital gene expression data. Bioinformatics. 2010;26:139–40.

14. Benjamini Y, Hochberg Y. Controlling the false discovery rate: a practical and powerful approach to multiple testing [Internet]. J. R. Stat. Soc. B. 1995. p. 289–300. Available from: http://www.stat.purdue.edu/~doerge/BIOINFORM.D/FALL06/Benjamini and Y FDR.pdf%5Cnhttp://engr.case.edu/ray_soumya/mlrg/controlling_fdr_benjamini95.pdf

15. Ward JH. Hierarchical Grouping to Optimize an Objective Function. J Am Stat Assoc. 1963;58:236–44.

16. Galili T. dendextend: An R package for visualizing, adjusting and comparing trees of hierarchical clustering. Bioinformatics. 2015;31:3718–20.

17. Maechler M, Rousseeuw P, Struyf A, Hubert M, Hornik K. Cluster Analysis Basics and Extensions. R package version 1.14.4. CRAN. 2013.

18. Warnes GR, Bolker B, Bonebakker L, Gentleman R, Huber W, Liaw A, Lumley T, Maechler M, Magnusson A, Moeller S, et al. gplots: Various R Programming Tools for Plotting Data. R Packag. version 2.13.0. 2014. p. 2015.

19. Wickham H. Elegant Graphics for Data Analysis. Media. 2009;35:211.

20. Wang J, Duncan D, Shi Z, Zhang B. WEB-based GEne SeT AnaLysis Toolkit (WebGestalt): update 2013. Nucleic Acids Res. 2013;41.

21. Gene Ontology Consortium. Gene Ontology Consortium: going forward. Nucleic Acids Res. 2015;43:D1049–56.

22. Gaudet P, Škunca N, Hu JC, Dessimoz C. Primer on the gene ontology. Methods Mol Biol. 2017. p. 25–37.

23. Supek F, Bošnjak M, Škunca N, Šmuc T. Revigo summarizes and visualizes long lists of gene ontology terms. PLoS One. 2011;6.

24. Kanehisa M, Goto S. Kyoto Encyclopedia of Genes and Genomes. Nucleic Acids Res [Internet]. 2000;28:27–30. Available from: http://www.genome.jp/kegg/

25. Durinck S, Moreau Y, Kasprzyk A, Davis S, De Moor B, Brazma A, et al. BioMart and Bioconductor: A powerful link between biological databases and microarray data analysis. Bioinformatics. 2005;21:3439–40.

26. Durinck S, Spellman PT, Birney E, Huber W. Mapping identifiers for the integration of genomic datasets with the R/ Bioconductor package biomaRt. Nat Protoc. 2009;4:1184–91.

27. Carlson M, Falcon S, Pages H, Li N. 2012. org. Mm. eg. db: Genome wide annotation for Mouse. *org Mm eg db Genome wide Annot Mouse*.

28. Luo W, Brouwer C. Pathview: An R/Bioconductor package for pathway-based data integration and visualization. Bioinformatics. 2013;29:1830–1.
