# Supplementary material for: Cycles of gene expression and genome response during mammalian tissue regeneration
Source: Epigenetics Chromatin. 2018 Sep 12;11:52. doi: 10.1186/s13072-018-0222-0 (PMC6134763; doi:10.1186/s13072-018-0222-0)
Supplement: Supplementary file 5 — Additional file 5: Figure S2. (A) Distribution of silhouette scores as a result of the PAM clustering of the varying gene expression Set 3 into 2–13 groups (k). The clustering into 7 groups was retained for our analyses in the main text (Fig. 2). In this Set 3.1 to 3.7 clustering, set 3.7 contained a large proportion of cell-cycle genes. The set in the k = 2–6 and 8–12 PAM clusterings most like Set 3.7 is labeled “3.7-like” in each case. For each clustering, the number of genes (nj) per group (j) is indicated to the right together with the average silhouette score (aveiϵCj Si). To the left of each clustering, the number of Set 3.7 genes from k = 7 in each group is indicated. Across clusterings, the highest average silhouette score is found for the most 3.7-like sets of genes. (B) Summary of the results of the functional enrichment analysis on the RNA-seq Set 1, Set 2 and Set 3 results. The GO terms displaying an enrichment p value lower than 10E − 10 were kept for analysis with the REVIGO tool. REVIGO aggregates synonymous GO terms and displays the aggregated terms as circles where the distance among circles indicates their similarity within the GO structure and their color indicates the associated p value, with blue signifying the lowest p values. Selected GO terms with highest p values are shown with the circle aggregates. Below, the highlighted GO terms have been listed with their associated p values with a log10 scale. (C) Gene-expression patterns post-PH in the KEGG cell-cycle pathway. The gene nodes in the KEGG cell-cycle pathway were colored using the “pathview” R package. Set 1 genes are colored gray, Set 2 genes are colored yellow, and Set 3 genes are displayed as a heat map that shows the relative transcript abundance between 0 h and 4 weeks post-PH from Fig. 2a. For the twenty-seven KEGG cell-cycle pathway nodes shared by multiple genes only the pattern for a representative gene (identified in Additional file 2: Figure S2d). (D) Selection of the represent [file 13072_2018_222_MOESM5_ESM.pdf]

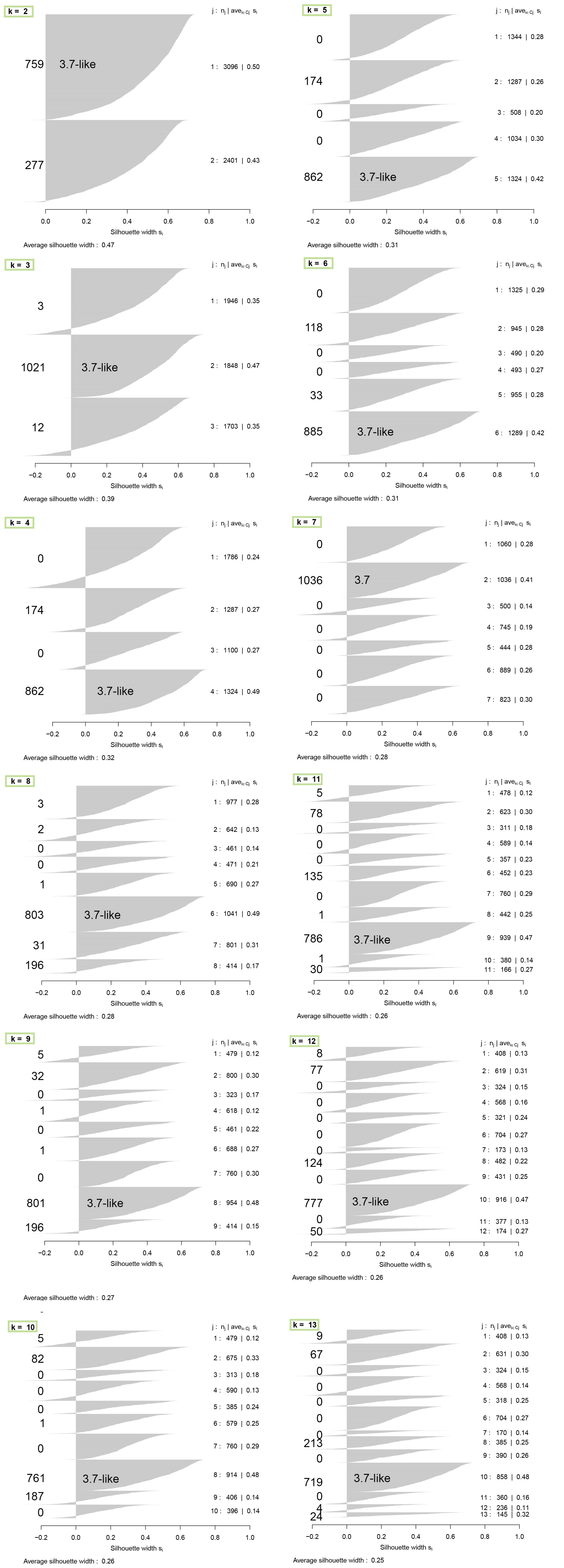

k = 2
759
3.7-like
277
1 : 3096 | 0.50
2 : 2401 | 0.43
Silhouette width s_i
Average silhouette width : 0.47
k = 5
0
174
0
0
862
3.7-like
1 : 1344 | 0.28
2 : 1287 | 0.26
3 : 508 | 0.20
4 : 1034 | 0.30
5 : 1324 | 0.42
Average silhouette width : 0.31
k = 3
3
1021
3.7-like
12
1 : 1946 | 0.35
2 : 1848 | 0.47
3 : 1703 | 0.35
Average silhouette width : 0.39
k = 6
0
118
0
0
33
885
3.7-like
1 : 1325 | 0.29
2 : 945 | 0.28
3 : 490 | 0.20
4 : 493 | 0.27
5 : 955 | 0.28
6 : 1289 | 0.42
Average silhouette width : 0.31
k = 4
0
174
0
862
3.7-like
1 : 1786 | 0.24
2 : 1287 | 0.27
3 : 1100 | 0.27
4 : 1324 | 0.49
Average silhouette width : 0.32
k = 7
0
1036
3.7
0
0
0
0
0
1 : 1060 | 0.28
2 : 1036 | 0.41
3 : 500 | 0.14
4 : 745 | 0.19
5 : 444 | 0.28
6 : 889 | 0.26
7 : 823 | 0.30
Average silhouette width : 0.28
k = 8
3
2
0
0
1
803
3.7-like
31
196
1 : 977 | 0.28
2 : 642 | 0.13
3 : 461 | 0.14
4 : 471 | 0.21
5 : 690 | 0.27
6 : 1041 | 0.49
7 : 801 | 0.31
8 : 414 | 0.17
Average silhouette width : 0.28
k = 11
5
78
0
0
0
135
0
1
786
3.7-like
1
30
1 : 478 | 0.12
2 : 623 | 0.30
3 : 311 | 0.18
4 : 589 | 0.14
5 : 357 | 0.23
6 : 452 | 0.23
7 : 760 | 0.29
8 : 442 | 0.25
9 : 939 | 0.47
10 : 380 | 0.14
11 : 166 | 0.27
Average silhouette width : 0.26
k = 9
5
32
0
1
0
1
0
801
3.7-like
196
1 : 479 | 0.12
2 : 800 | 0.30
3 : 323 | 0.17
4 : 618 | 0.12
5 : 461 | 0.22
6 : 688 | 0.27
7 : 760 | 0.30
8 : 954 | 0.48
9 : 414 | 0.15
Average silhouette width : 0.27
k = 12
8
77
0
0
0
0
0
124
0
777
3.7-like
0
50
1 : 408 | 0.13
2 : 619 | 0.31
3 : 324 | 0.15
4 : 568 | 0.16
5 : 321 | 0.24
6 : 704 | 0.27
7 : 173 | 0.13
8 : 482 | 0.22
9 : 431 | 0.25
10 : 916 | 0.47
11 : 377 | 0.13
12 : 174 | 0.27
Average silhouette width : 0.26
k = 10
5
82
0
0
0
1
0
761
3.7-like
187
0
1 : 479 | 0.12
2 : 675 | 0.33
3 : 313 | 0.18
4 : 590 | 0.13
5 : 385 | 0.24
6 : 579 | 0.25
7 : 760 | 0.29
8 : 914 | 0.48
9 : 406 | 0.14
10 : 396 | 0.14
Average silhouette width : 0.26
k = 13
9
67
0
0
0
0
0
213
0
719
3.7-like
0
4
24
1 : 408 | 0.13
2 : 631 | 0.30
3 : 324 | 0.15
4 : 568 | 0.14
5 : 318 | 0.25
6 : 704 | 0.27
7 : 170 | 0.14
8 : 385 | 0.25
9 : 390 | 0.26
10 : 858 | 0.48
11 : 360 | 0.16
12 : 236 | 0.11
13 : 145 | 0.32
Average silhouette width : 0.25

Sup. Fig. 2A

Set 1

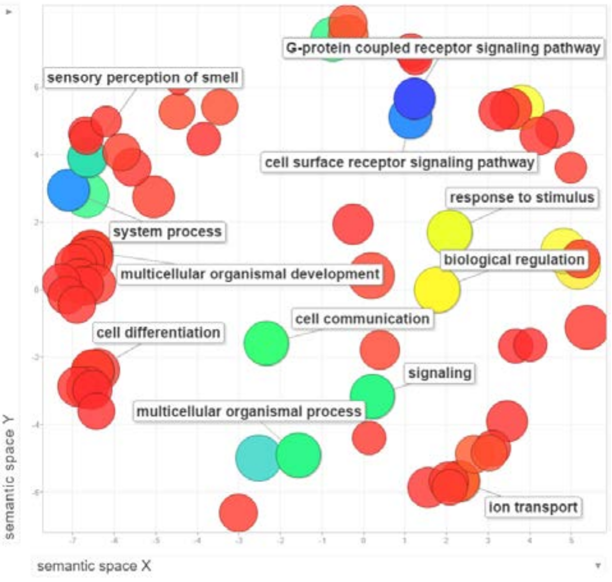

Set 2

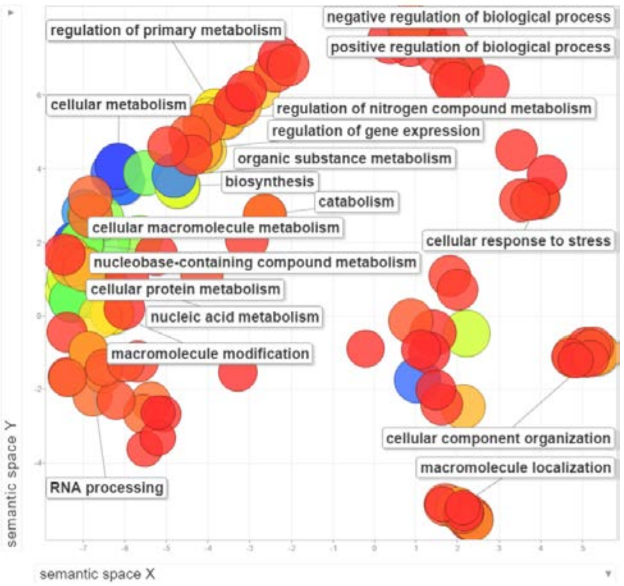

Set 3

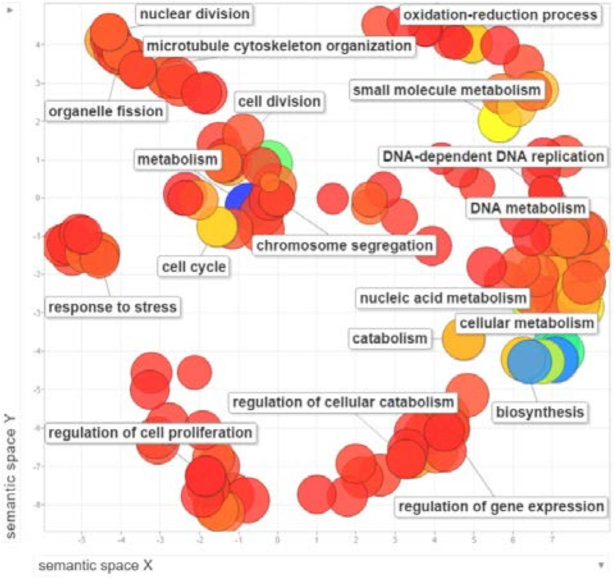

Set 1 GO terms	p-values [log10]
G-protein coupled receptor signaling pathway	-300
Cell surface receptor signaling pathway	-266
System process	-261
Sensory perception of smell	-222
Multicellular organismal process	-208
Signaling	-207
Single-multicellular organism process	-206
Cell communication	-200
Response to stimulus	-119
Biological regulation	-105
Ion transport	-41
Multicellular organismal development	-22
Cell differentiation	-21

Set 2 GO terms	p-values [log10]
Cellular metabolism	-268
Cellular macromolecule metabolism	-260
Organic substance metabolism	-249
Cellular protein metabolism	-163
Nucleobase-containing compound metabolism	-158
Nucleic acid metabolism	-139
Biosynthesis	-115
Macromolecule modification	-90
Regulation of primary metabolism	-89
Regulation of nitrogen compound metabolism	-71
Regulation of gene expression	-67
Macromolecule localization	-63
Cellular component organization	-62
RNA processing	-61
Catabolism	-46
Positive regulation of biological process	-30
Cellular response to stress	-28
Negative regulation of biological process	-27

Set 3 GO terms	p-values [log10]
Cellular metabolism	-219
Organic substance metabolism	-184
Small molecule metabolism	-90
Nucleobase-containing compound metabolism	-82
Cell cycle	-76
Oxidation-Reduction process	-75
Biosynthesis	-74
Catabolism	-68
Nucleic acid metabolism	-60
Cellular component organization	-57
Regulation of primary metabolism	-48
Negative regulation of biological process	-42
Positive regulation of biological process	-41
DNA metabolism	-40
Response to stress	-40
Cell division	-39
Cellular response to stress	-39
Organelle fission	-34
Nuclear division	-33
Reg. gene expression	-25
Microtubule cytoskeleton organization	-24
Chromosome segregation	-21
Regulation cellular catabolism	-17
DNA-dependent DNA replication	-16
Regulation of cell proliferation	-12

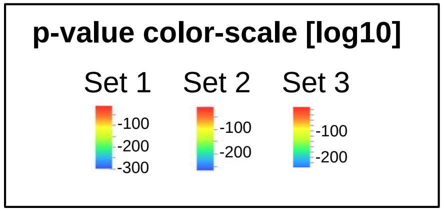

Sup. Fig. 2B

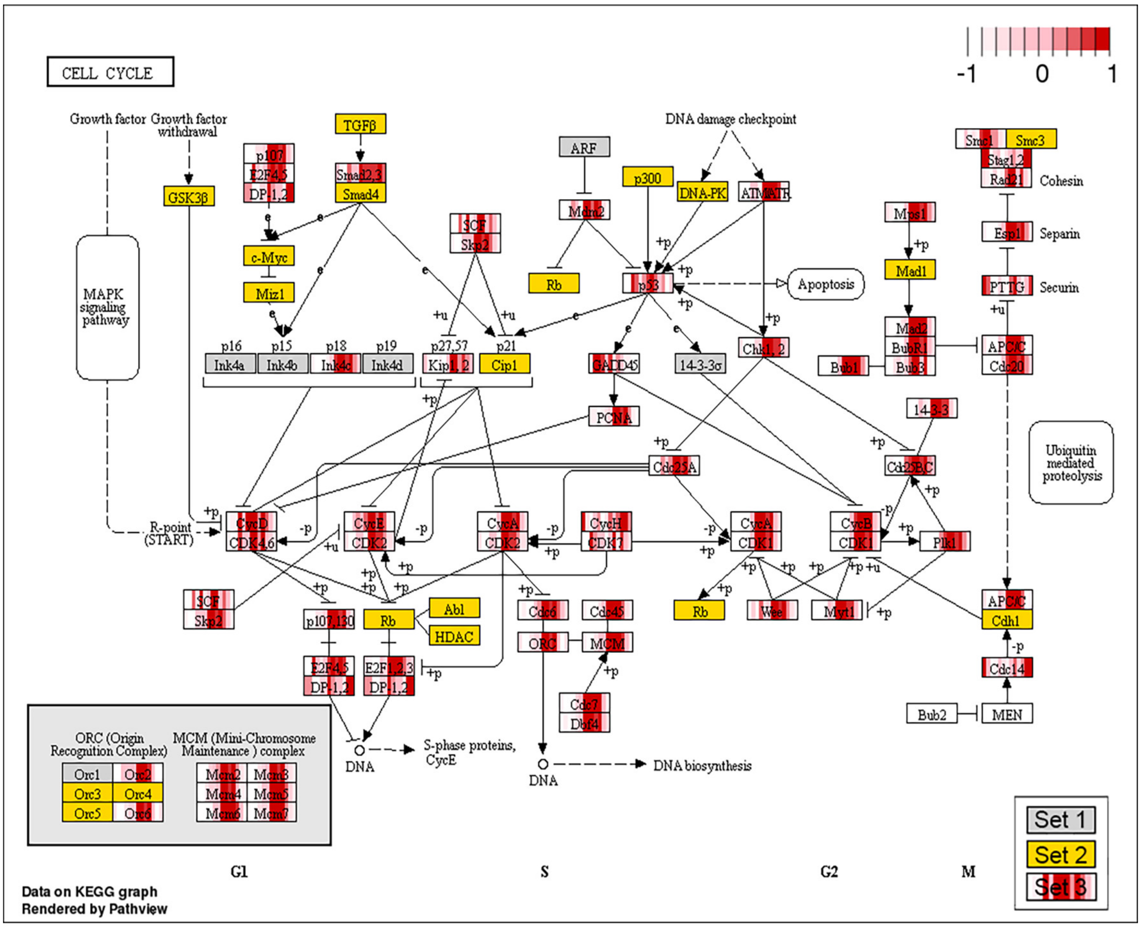
CELL CYCLE
-1 0 1
Data on KEGG graph
Rendered by Pathview
Set 1
Set 2
Set 3

Sup. Fig. 2C

Node	Gene 1	Gene 2	Gene 3	Gene 4	Gene 5	Gene 6	Gene 7	Gene 8	Gene 9	Gene 10	Gene 11	Gene 12
**DP1,2**	Tfdp1 (3.6)	Tfdp2 (3.1)										
**E2F4,5**	E2f4 (3.5)	E2f5 (2)										
**Kip1,2**	Cdkn1b (3.7)	Cdkn1c (2)										
**Chk1,2**	Chek1 (3.7)	Chek2 (3.7)										
**Stag1,2**	Stag1 (3.6)	Stag2 (3.1)										
**TGFB**	Tgfb1 (2)	Tgfb2 (1)	Tgfb3 (1)									
**SCF**	Gm9840 (1)	Skp1a (2)	Cul1 (3.5)	Rbx1 (2)								
**p300**	Ep300 (2)	Crebbp (2)										
**ATM-ATR**	Atm (2)	Atr (3.6)										
**GADD45**	Gadd45a (3.2)	Gadd45b (3.4)	Gadd45g (3.4)									
**Mad2**	Mad2l1 (3.7)	Mad2l2 (3.1)										
**Smc1**	Smc1a (3.7)	Smc1b (1)										
**APC/C**	Anapc1 (2)	Cdc27 (3.7)	Anapc4 (3.6)	Cdc23 (2)	Anapc7 (2)	Anapc5 (3.7)	Anapc11 (3.7)	Cdc26 (2)	Anapc10 (2)	Anapc13 (3.7)	Cdc16 (2)	Anapc2 (2)
**14-3-3**	Ywhae (2)	Ywhag (2)	Ywhah (3.7)	Ywhaq (2)	Ywhaz (2)	Ywhab (2)						
**Cdc25b,c**	Cdc25b (3.7)	Cdc25c (3.7)										
**Cdc14**	Cdc14a (1)	Cdc14b (3.1)										
**Cycb**	Ccnb1 (3.7)	Ccnb2 (3.7)	Ccnb3 (1)									
**Wee**	Wee1 (3.6)	Wee2 (1)										
**Cyca**	Ccna1 (1)	Ccna2 (3.7)										
**MCM**	Mcm2 (3.7)	Mcm3 (3.7)	Mcm3 (3.7)	Mcm4 (3.7)	Mcm5 (3.7)	Mcm6 (3.7)	Mcm7 (3.7)					
**ORC**	Orc1 (1)	Orc2 (3.5)	Orc3 (2)	Orc4 (2)	Orc5 (2)	Orc6 (3.7)						
**HDAC**	Hdac1 (2)	Hdac2 (2)										
**E2f1,2,3**	E2f1 (3.7)	E2f2 (3.7)	E2f3 (3.6)									
**p107,p130**	Rbl1 (3.7)	Rbl2 (2)										
**CycE**	Ccne1 (3.5)	Ccne2 (3.6)										
**CDK4,6**	Cdk4 (3.6)	Cdk6 (2)										
**CycD**	Ccnd1 (3.6)	Ccnd2 (2)	Ccnd3 (3.6)									

Sup. Fig. 2D
