# Supplementary figures and images for: Cycles of gene expression and genome response during mammalian tissue regeneration
Source: Epigenetics Chromatin. 2018 Sep 12;11:52. doi: 10.1186/s13072-018-0222-0 (PMC6134763; doi:10.1186/s13072-018-0222-0)

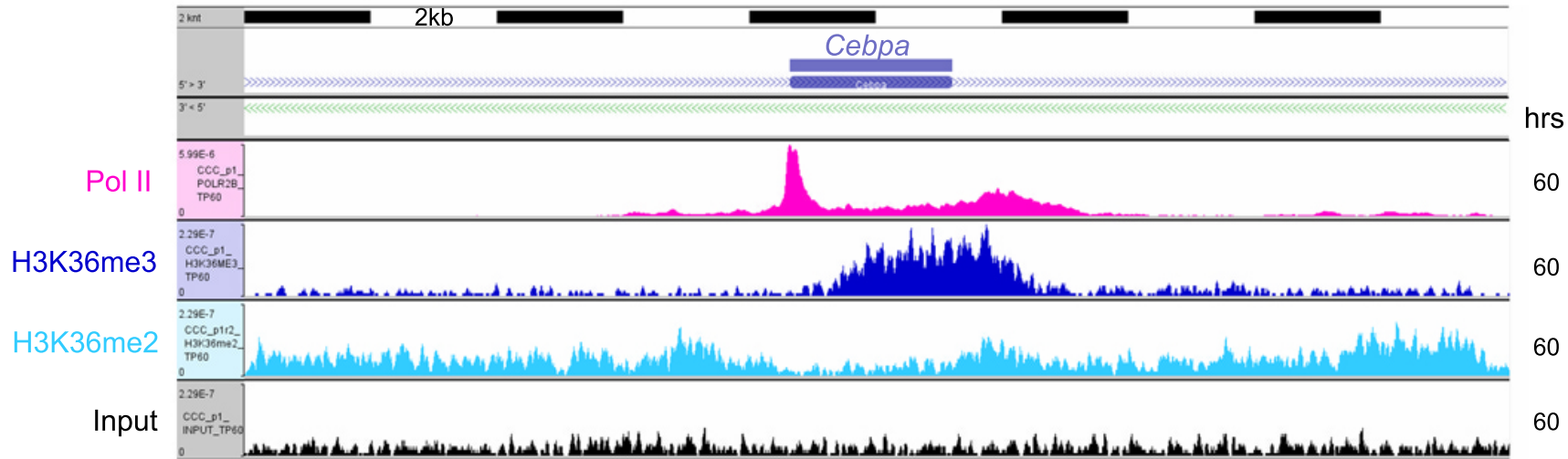

Sup. Fig. 4

Supplement: Supplementary file 7 — Additional file 7: Figure S4. Pol II, H3K36me2 and H3K36me3 densities at 60 h post-PH for the Cebpa gene. (A) Genomic view of the Cebpa gene. Densities of the central 50 bp of paired-end reads for Pol II (pink), H3K36me3 (dark blue) and H3K36me2 (light blue) ChIP fragments are shown for 60 h post-PH. Similarly, input fragments at 60 h post-PH are shown. [file 13072_2018_222_MOESM7_ESM.pdf]

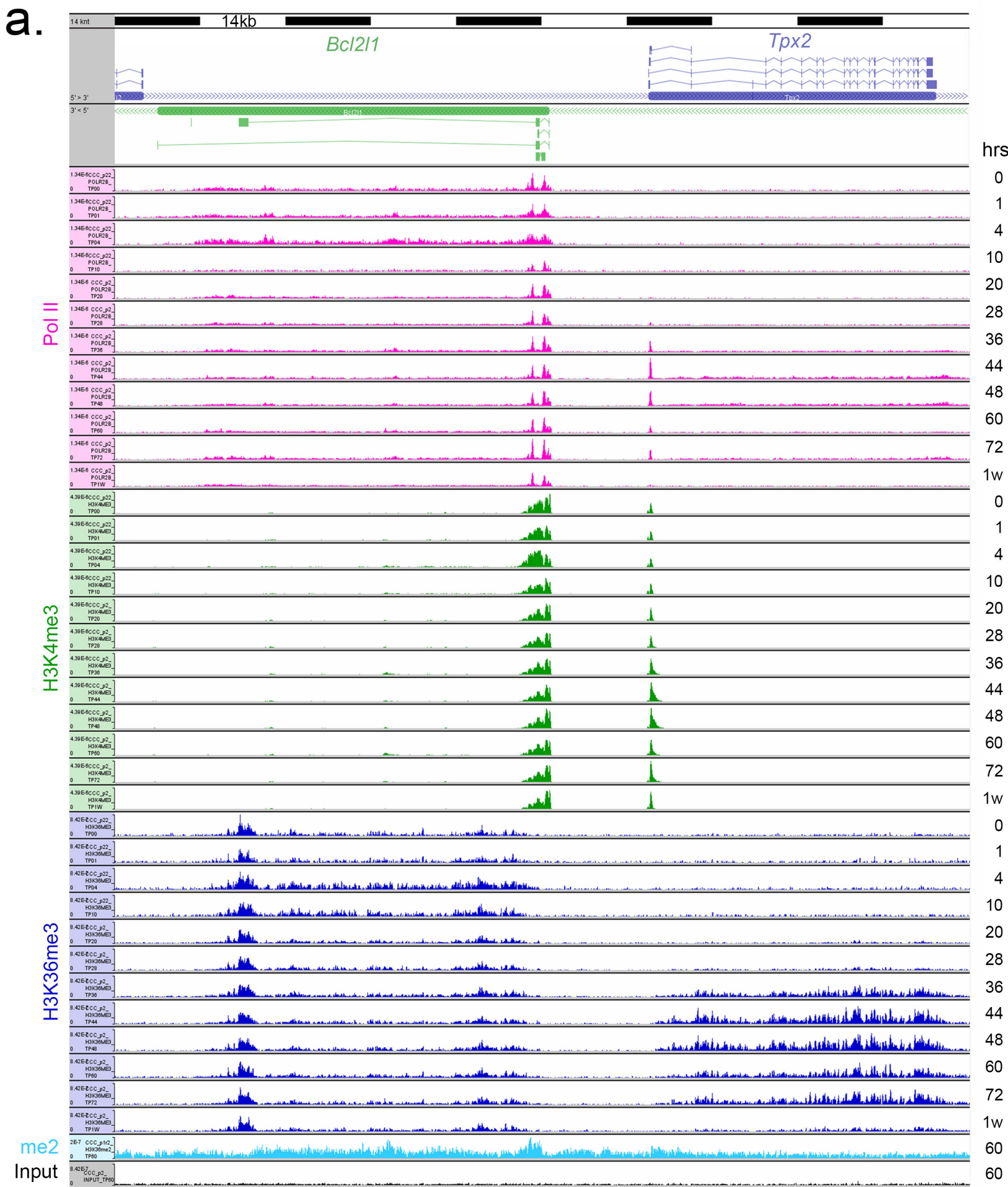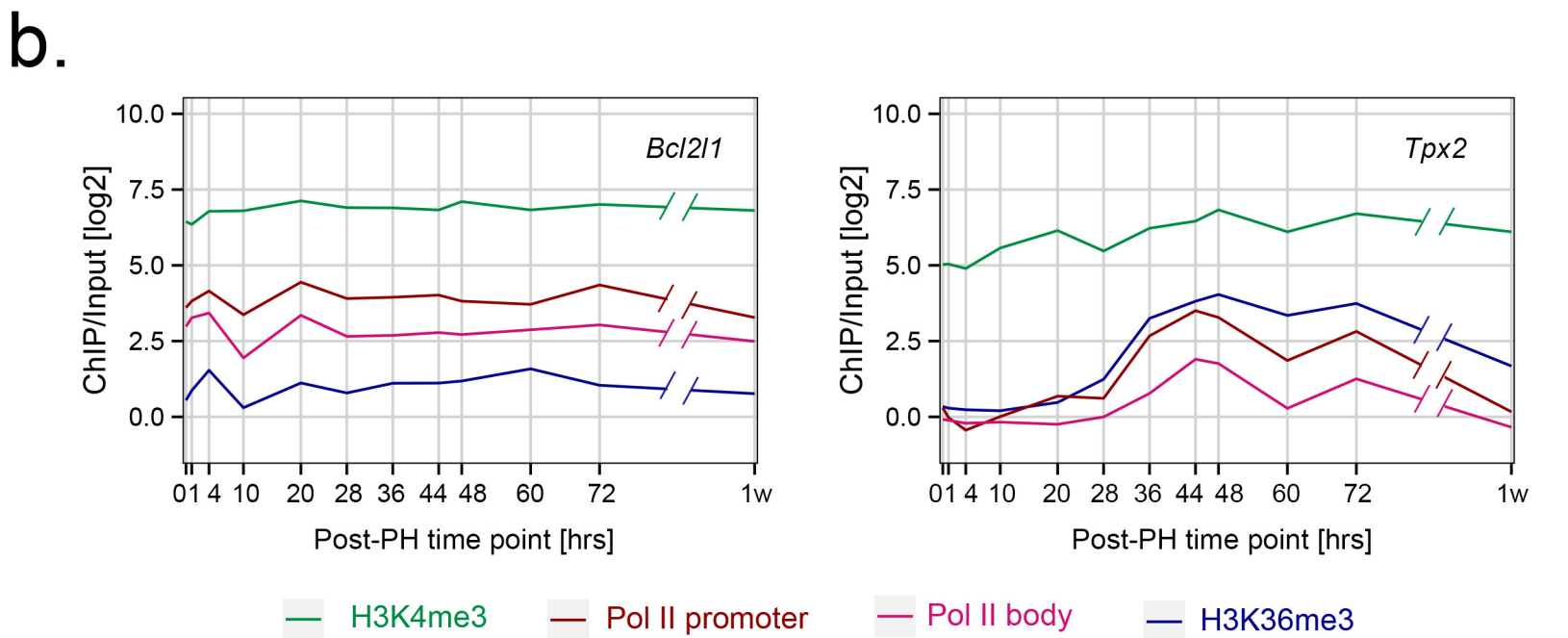

Sup. Fig. 3A

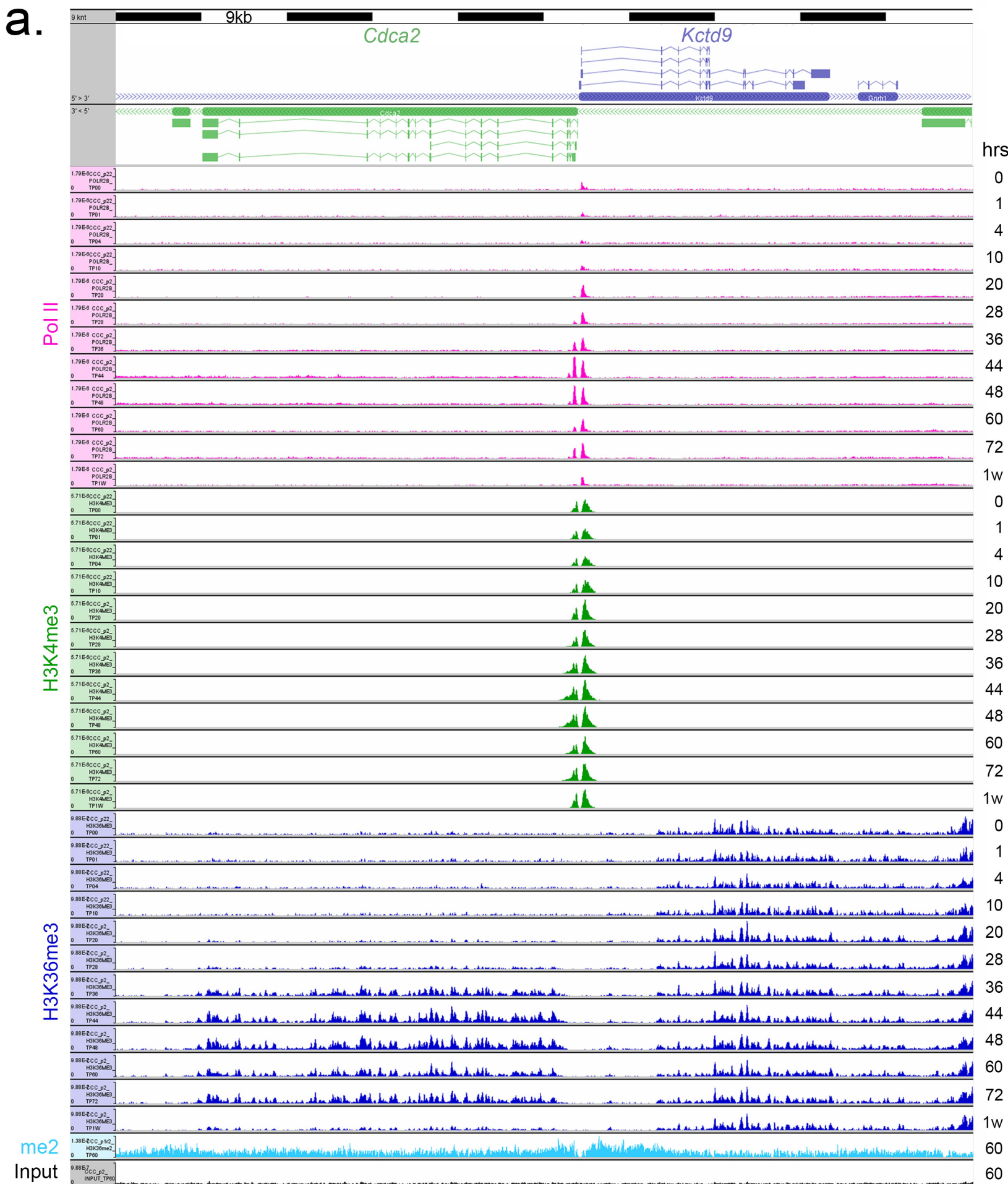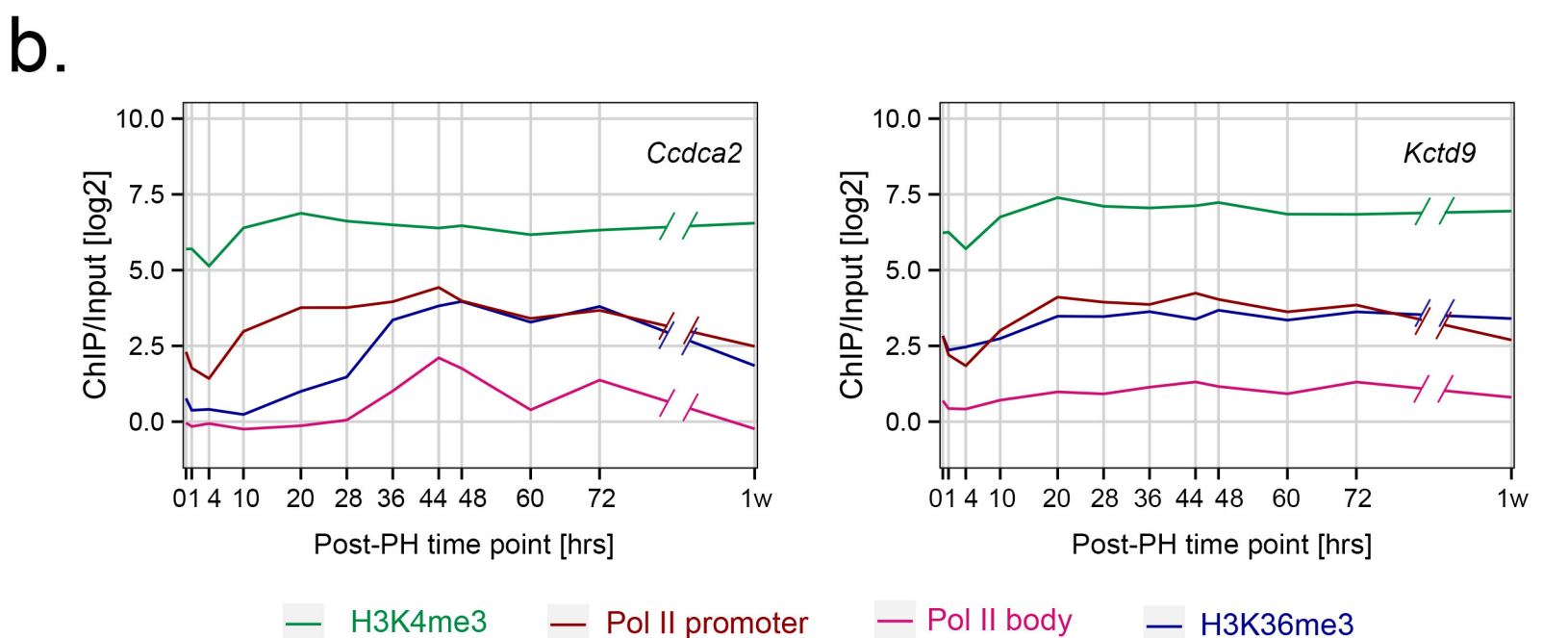

Sup. Fig. 3B

a.

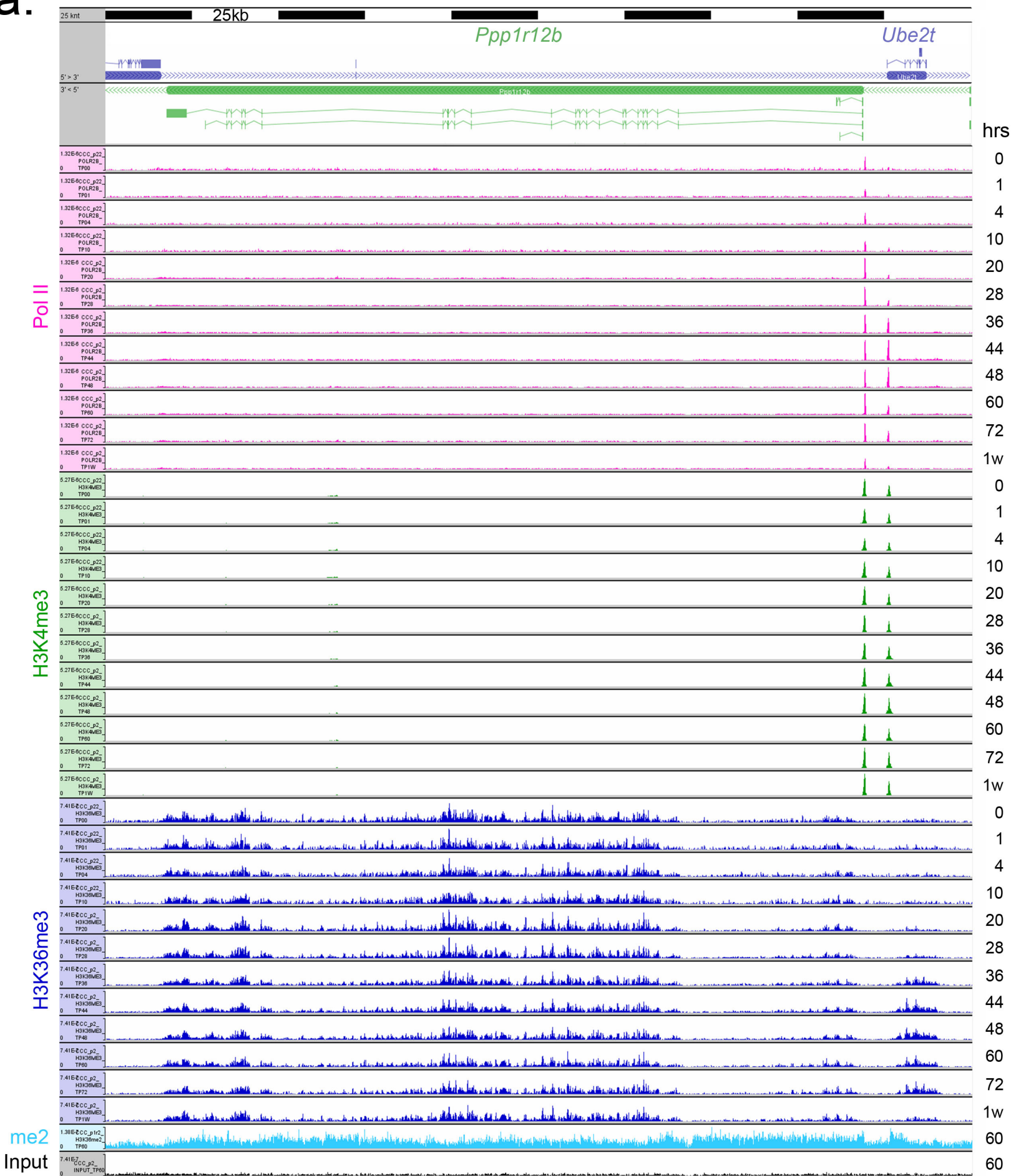

b.

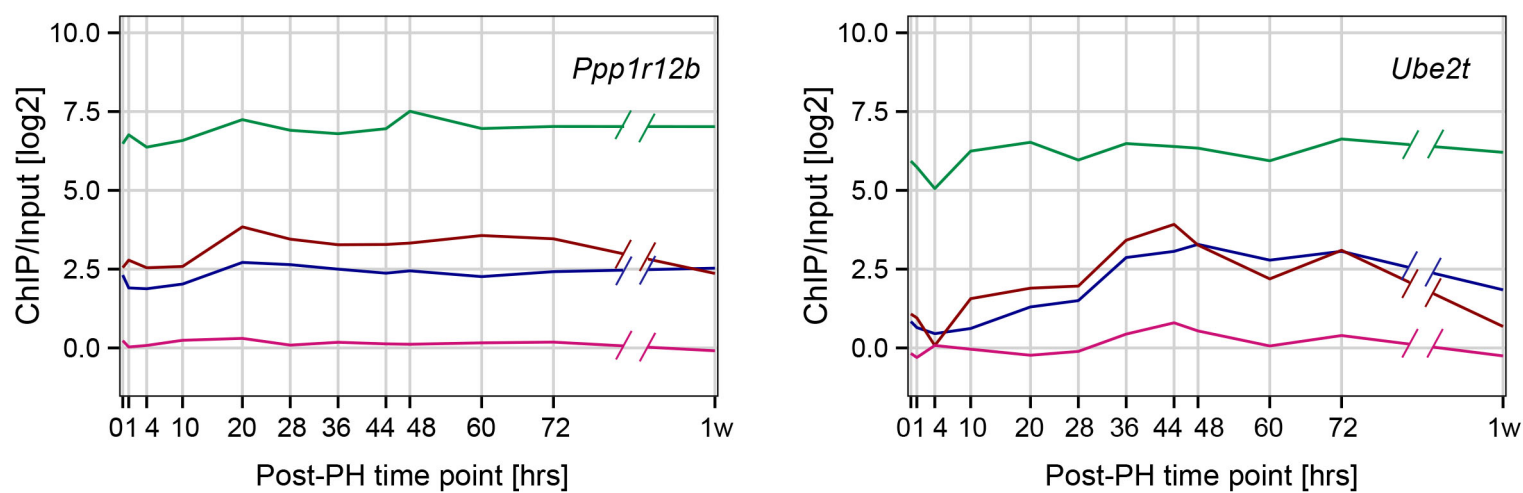

Sup. Fig. 3C

a.

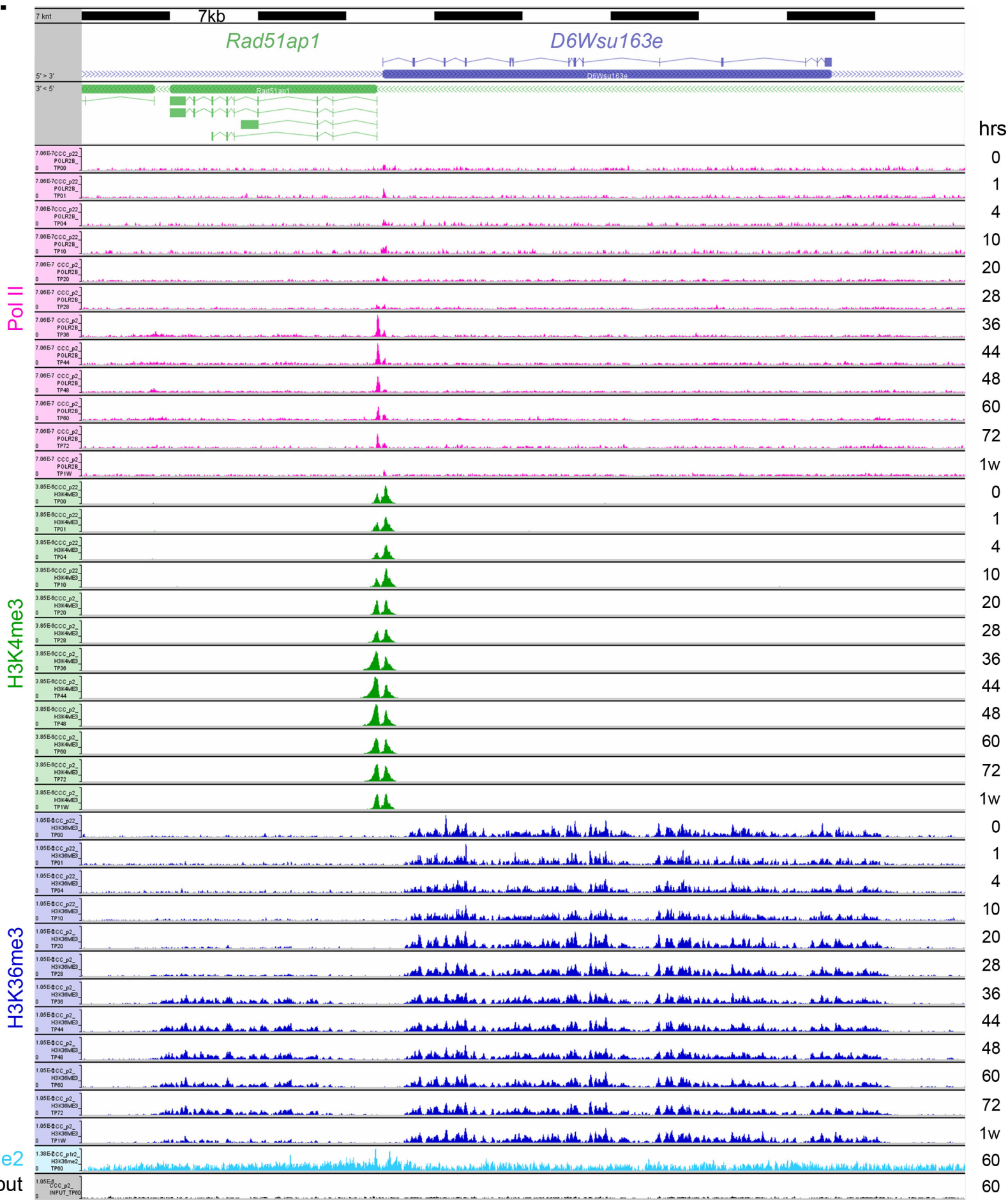

b.

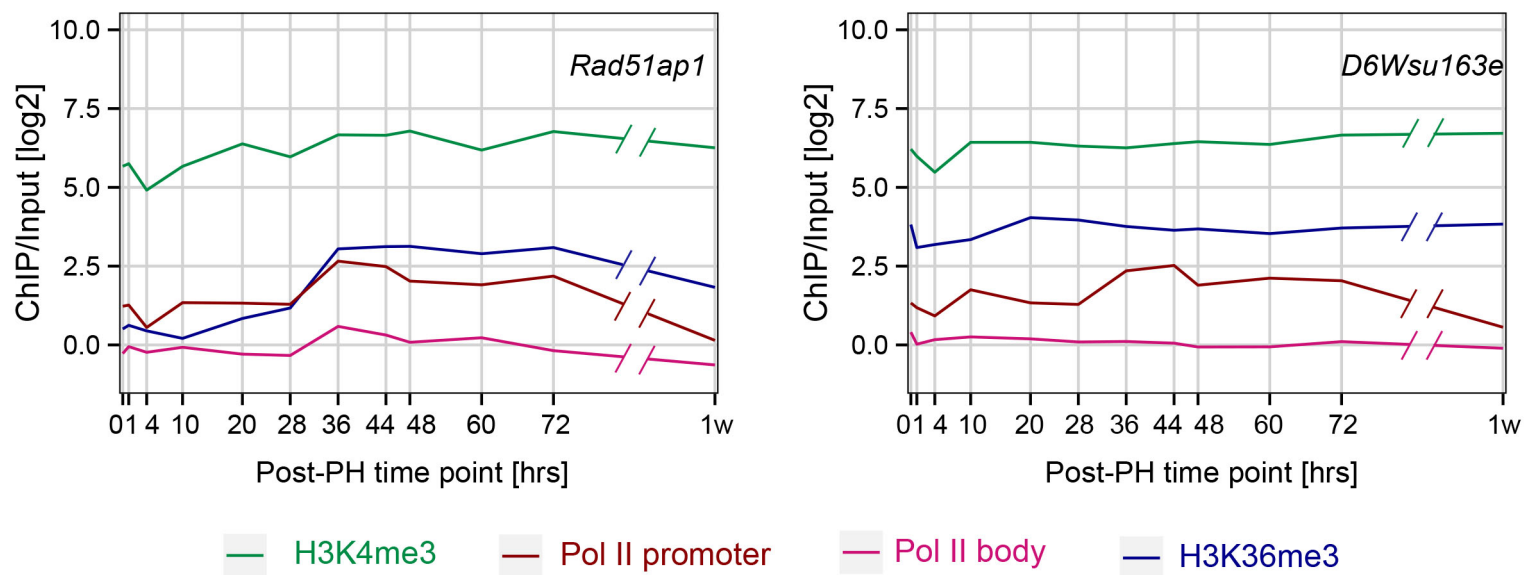

Sup. Fig. 3D

a.

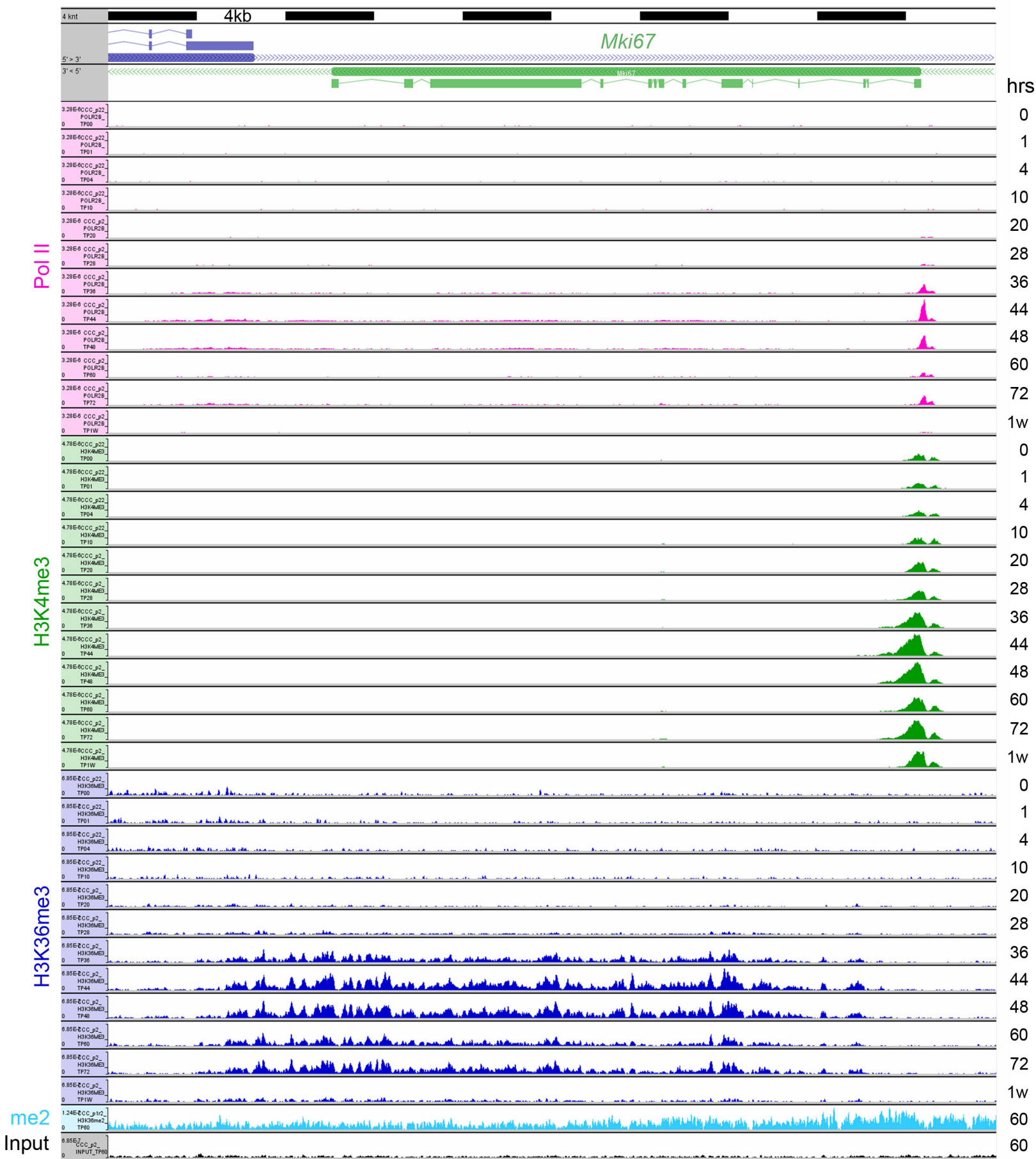

b.

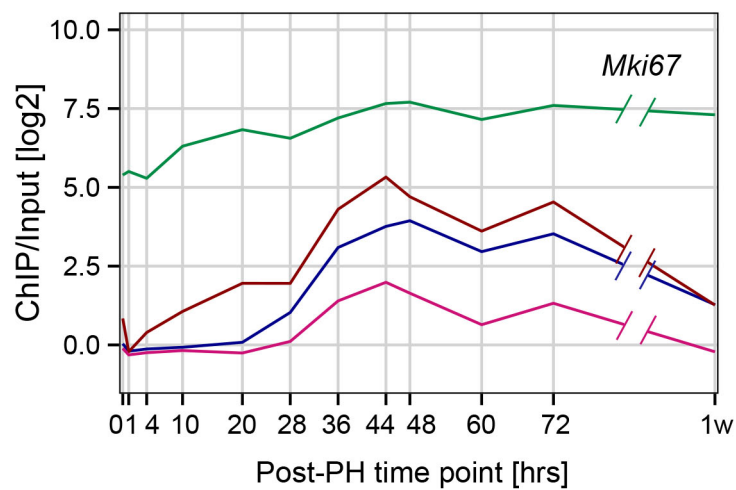

— H3K4me3 — Pol II promoter — Pol II body — H3K36me3

Sup. Fig. 3E

Supplement: Supplementary file 9 — Additional file 9: Figures S3. (A–E) Additional examples of transcriptional gene activity post-PH. Displays of the ChIP-Seq results of five different gene sets—(A) Bcl2l1 and Tpx2; (B) Cdca2 and Kctd9; (C) mKi67; (D) Ppp1r12b and Ube2t; and (E) Rad51ap1 and D6Wsu163e—are shown as described in Fig. 3. [file 13072_2018_222_MOESM9_ESM.pdf]

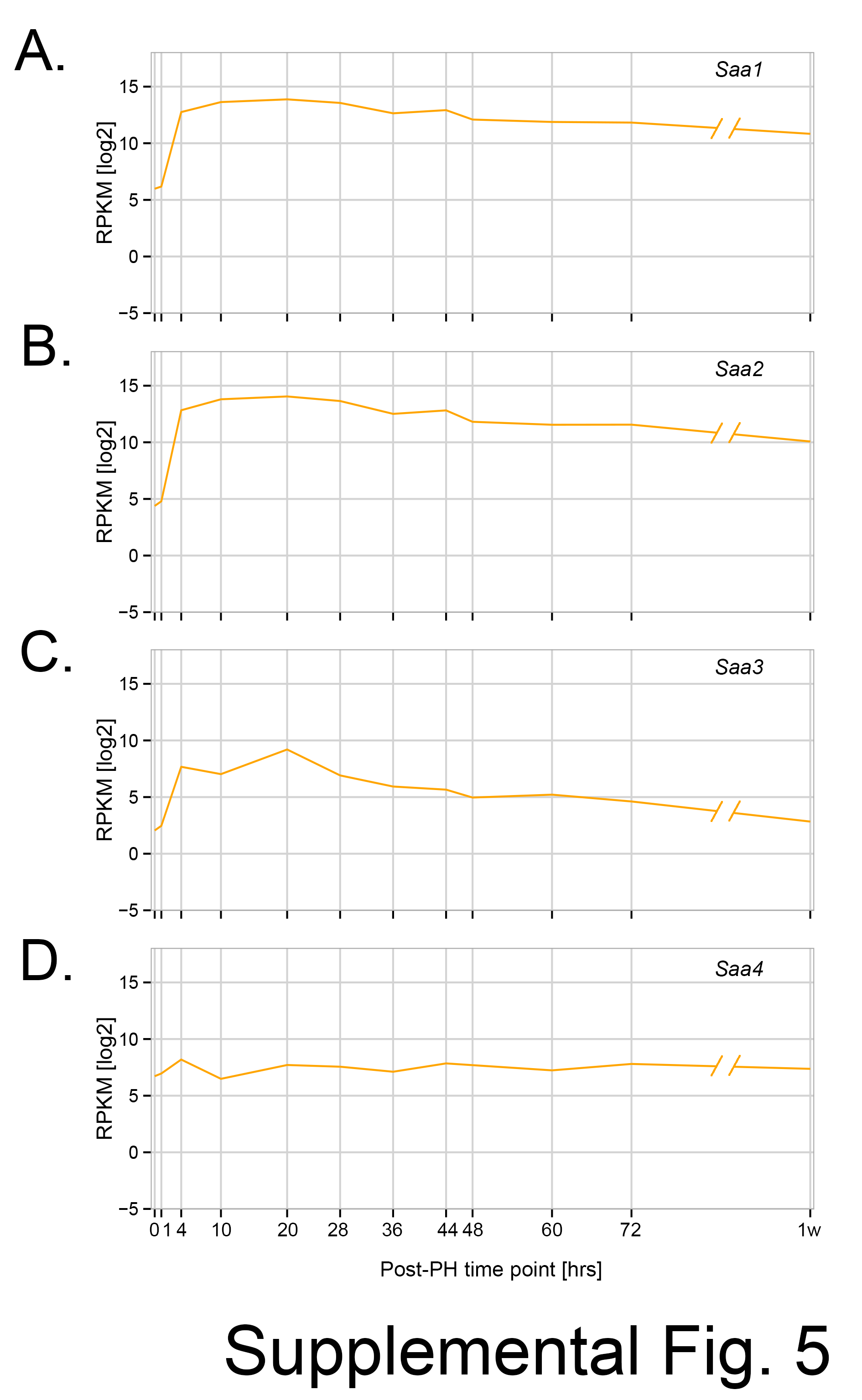

Supplement: Supplementary file 10 — Additional file 10: Figure S5. RNA abundance of the acute-response Saa genes post-PH. RNA-seq RPKM values for the (A) Saa1, (B) Saa2, (C) Saa3, and (D) Saa4 genes (top-to-bottom) post-PH. [file 13072_2018_222_MOESM10_ESM.tif]

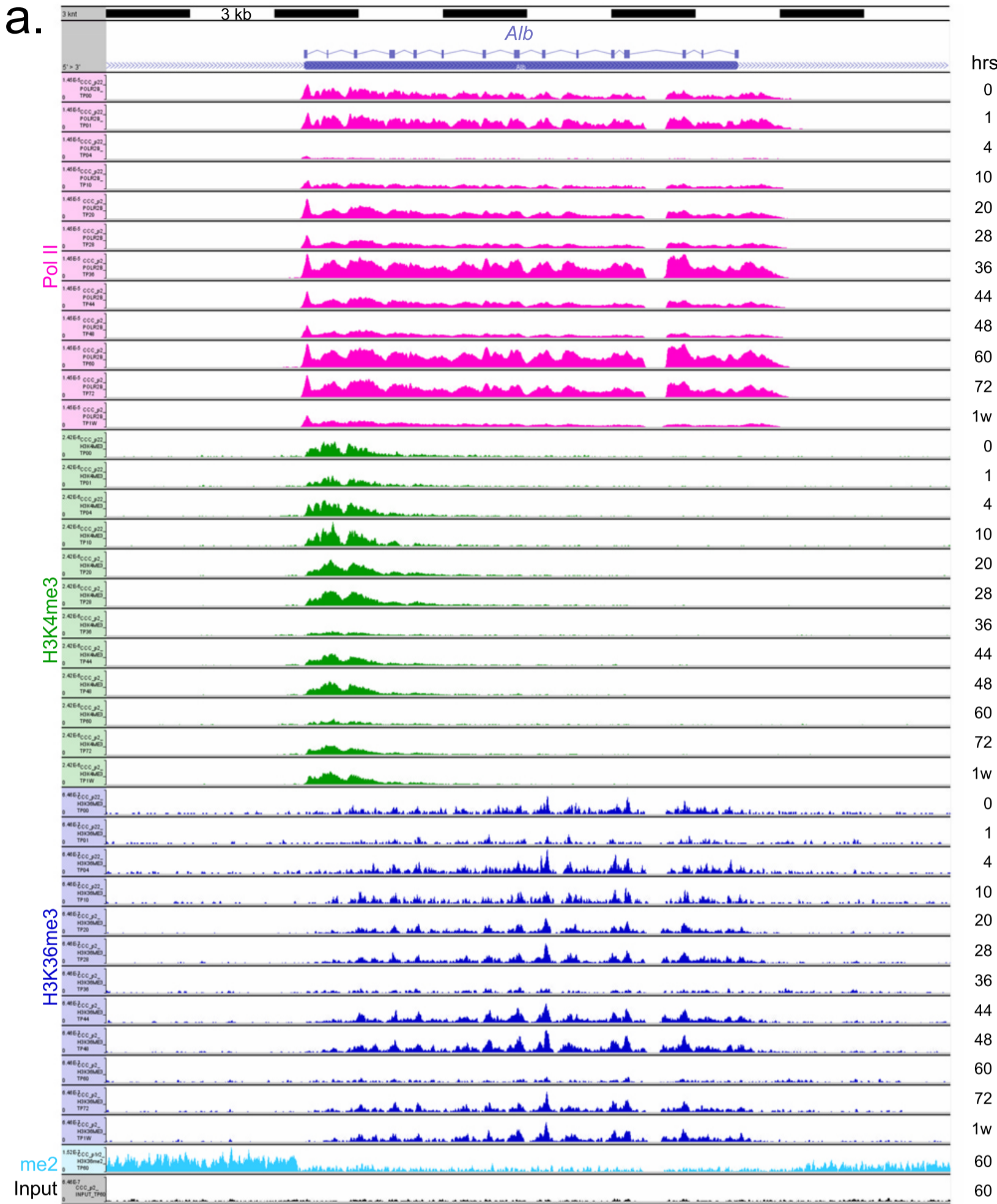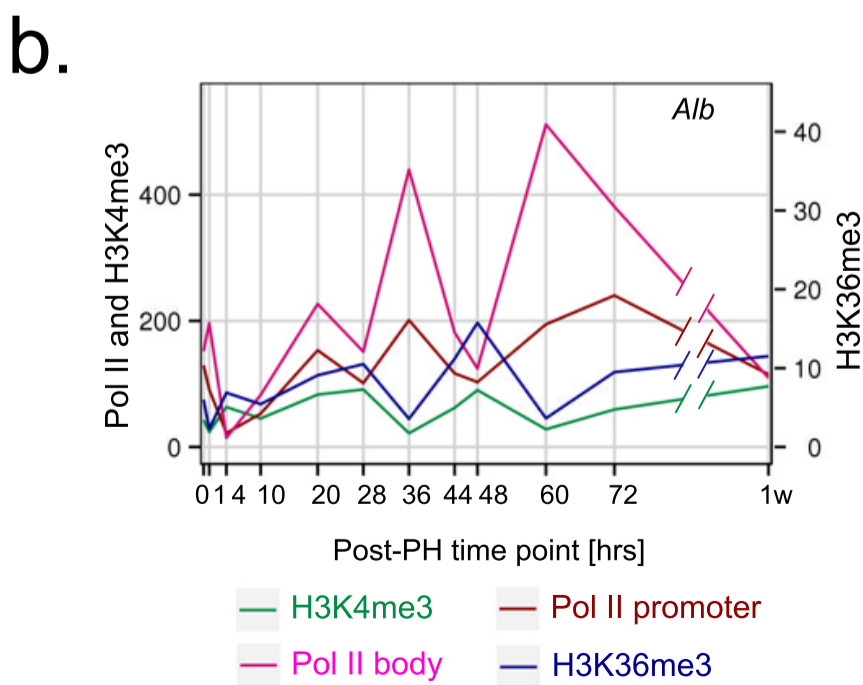

Sup. Fig. 6

Supplement: Supplementary file 11 — Additional file 11: Figure S6. Pol II, H3K36me2 and H3K36me3 densities at 60 h post-PH for the Alb gene. (A) Genomic view of the Alb gene. Densities of the central 50 bp of paired-end reads for Pol II (pink), H3K4me3 (green) and H3K36me3 (blue) ChIP fragments are shown for 0 h to 1 week post-PH. Similarly, densities for H3K36me2 and input fragments at 60 h post-PH are shown. (B) H3K4me3 (green), Pol II promoter (burgundy) and body (pink), and H3K36me3 (blue) log2 ChIP/input fragment-density comparison for the Alb gene post-PH. The central 50 bp of the paired-end reads were used for quantification. The regions used for each quantitation are given in the text. [file 13072_2018_222_MOESM11_ESM.pdf]
